# (*E*)-*N*-[(6-Bromo­pyridin-2-yl)methyl­idene]-4-methyl­aniline

**DOI:** 10.1107/S1600536811031825

**Published:** 2011-08-17

**Authors:** Mingjian Cai, Penggao Ma, Xiuge Wang, Tao Sun

**Affiliations:** a Department of Chemistry, Tangshan Normal University, Tangshan 063000, People’s Republic of China; b Lanzhou Petrochemical Research Center, PetroChina Lanzhou, Gansu 300072, People’s Republic of China

## Abstract

The title compound, C_13_H_11_BrN_2_, a Schiff base obtained from 6-bromo­picolinaldehyde and *p*-toluidine, has an *E* configuration about the C=N bond. The dihedral angle between the benzene and pyridine rings is 30.4 (1)°.

## Related literature

For Schiff base complexes with transition metals, see: Burkhardt & Plass (2008 ▶); Keypour *et al.* (2011 ▶); Tarafder *et al.* (2002 ▶). For their complexing ability towards toxic metals, see: Kocyigit *et al.* (2010 ▶);
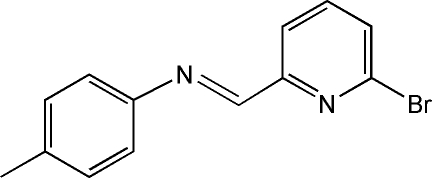


## Experimental

### 

#### Crystal data

C_13_H_11_BrN_2_
*M* *_r_* = 275.15
Orthorhombic, 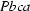

*a* = 13.542 (3) Å
*b* = 6.1544 (15) Å
*c* = 27.620 (7) Å
*V* = 2301.9 (10) Å^3^
*Z* = 8
Mo *K*α radiation
μ = 3.54 mm^−1^
*T* = 113 K
0.20 × 0.08 × 0.04 mm

#### Data collection

Rigaku Saturn724 CCD diffractometer
Absorption correction: multi-scan (*CrystalClear*; Rigaku/MSC, 2002 ▶) *T*_min_ = 0.538, *T*_max_ = 0.871
21379 measured reflections
2750 independent reflections
2251 reflections with *I* > 2σ(*I*)
*R*_int_ = 0.044

#### Refinement

*R*[*F* ^2^ > 2σ(*F* ^2^)] = 0.040
*wR*(*F* ^2^) = 0.104
*S* = 1.08
2750 reflections
146 parameters
H-atom parameters constrained
Δρ_max_ = 0.91 e Å^−3^
Δρ_min_ = −0.66 e Å^−3^

### 

Data collection: *CrystalClear* (Rigaku/MSC, 2002 ▶); cell refinement: *CrystalClear*; data reduction: *CrystalClear*; program(s) used to solve structure: *SHELXS97* (Sheldrick, 2008 ▶); program(s) used to refine structure: *SHELXL97* (Sheldrick, 2008 ▶); molecular graphics: *DIAMOND* (Crystal Impact, 2009 ▶); software used to prepare material for publication: *CrystalStructure* (Rigaku/MSC, 2006 ▶).

## Supplementary Material

Crystal structure: contains datablock(s) I, global. DOI: 10.1107/S1600536811031825/ld2022sup1.cif

Structure factors: contains datablock(s) I. DOI: 10.1107/S1600536811031825/ld2022Isup2.hkl

Supplementary material file. DOI: 10.1107/S1600536811031825/ld2022Isup3.cml

Additional supplementary materials:  crystallographic information; 3D view; checkCIF report

## Figures and Tables

**Fig. 1.**
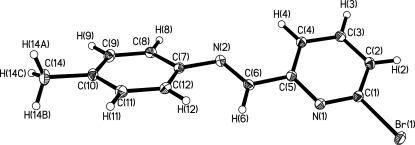
The molecular structure of the title compound, showing 30% probability displacement ellipsoids and the atom-numbering scheme.

## supplementary crystallographic information

### Comment

Schiff bases have played an important role in the development of coordination chemistry as they readily form stable complexes with most of the transition metals (Burkhardt & Plass, 2008; Keypour, *et al.*, 2011; Tarafder, *et al.*, 2002). They show important properties, *e.g.* an ability to reversibly bind oxygen, catalytic activity in hydrogenation of olefins, transfer of amino group, photochromic properties and complexing ability towards toxic metals (Kocyigit *et al.*, 2010). In this paper, the structure of the new Schiff base derived from condensation of 6-bromopicolinaldehyde with *p*-toluidine is reported. The molecule of the title compound, Fig. 1, possesses an E configuration about the C6=N2 bond.

### Experimental

The solution of 6-bromopicolinaldehyde and *p*-toluidine in methanol was refluxed for 2 h, and then the crude product was isolated by filtration and recrystallized from methanol to yield yellowish title compound. Finally, the title compound was dissolved in a small amount of methanol and the solution was kept for 5 days at ambient temperature to give rise to yellowish block-like crystals on slowly evaporating the solvent.

### Refinement

The hydrogen atoms were positioned geometrically (C—H=0.93–0.98 Å) and refined using a riding model, with *U*_iso_(H)=1.2 or 1.5*U*_eq_(C) (methyl group). The methyl group position was rotationally optimized (AFIX 137)

### Crystal data

**Table:**

C_13_H_11_BrN_2_	*D*_x_ = 1.588 Mg m^−^^3^
*M**_r_* = 275.15	Mo *K*α radiation, λ = 0.71073 Å
Orthorhombic, *P**b**c**a*	Cell parameters from 6762 reflections
*a* = 13.542 (3) Å	θ = 2.1–28.0°
*b* = 6.1544 (15) Å	µ = 3.54 mm^−^^1^
*c* = 27.620 (7) Å	*T* = 113 K
*V* = 2301.9 (10) Å^3^	Prism, colorless
*Z* = 8	0.20 × 0.08 × 0.04 mm
*F*(000) = 1104	

### Data collection

**Table:**

Rigaku Saturn724 CCD diffractometer	2750 independent reflections
Radiation source: rotating anode	2251 reflections with *I* > 2σ(*I*)
multilayer	*R*_int_ = 0.044
Detector resolution: 14.22 pixels mm^-1^	θ_max_ = 27.9°, θ_min_ = 2.1°
ω and φ scans	*h* = −17→17
Absorption correction: multi-scan (*CrystalClear*; Rigaku/MSC, 2002)	*k* = −7→8
*T*_min_ = 0.538, *T*_max_ = 0.871	*l* = −36→36
21379 measured reflections	

### Refinement

**Table:**

Refinement on *F*^2^	Primary atom site location: structure-invariant direct methods
Least-squares matrix: full	Secondary atom site location: difference Fourier map
*R*[*F*^2^ > 2σ(*F*^2^)] = 0.040	Hydrogen site location: inferred from neighbouring sites
*wR*(*F*^2^) = 0.104	H-atom parameters constrained
*S* = 1.08	*w* = 1/[σ^2^(*F*_o_^2^) + (0.0529*P*)^2^] where *P* = (*F*_o_^2^ + 2*F*_c_^2^)/3
2750 reflections	(Δ/σ)_max_ = 0.002
146 parameters	Δρ_max_ = 0.91 e Å^−^^3^
0 restraints	Δρ_min_ = −0.66 e Å^−^^3^

### Special details

**Table:**

Geometry. All e.s.d.'s (except the e.s.d. in the dihedral angle between two l.s. planes) are estimated using the full covariance matrix. The cell e.s.d.'s are taken into account individually in the estimation of e.s.d.'s in distances, angles and torsion angles; correlations between e.s.d.'s in cell parameters are only used when they are defined by crystal symmetry. An approximate (isotropic) treatment of cell e.s.d.'s is used for estimating e.s.d.'s involving l.s. planes.
Refinement. Refinement of *F*^2^ against ALL reflections. The weighted *R*-factor *wR* and goodness of fit *S* are based on *F*^2^, conventional *R*-factors *R* are based on *F*, with *F* set to zero for negative *F*^2^. The threshold expression of *F*^2^ > σ(*F*^2^) is used only for calculating *R*-factors(gt)*etc*. and is not relevant to the choice of reflections for refinement. *R*-factors based on *F*^2^ are statistically about twice as large as those based on *F*, and *R*-factors based on ALL data will be even larger.

### Fractional atomic coordinates and isotropic or equivalent isotropic displacement parameters (Å^2^)

**Table:**

	*x*	*y*	*z*	*U*_iso_*/*U*_eq_	
Br1	0.38420 (2)	0.10293 (5)	0.750452 (7)	0.03018 (13)	
N1	0.38367 (13)	0.1724 (3)	0.65201 (6)	0.0218 (4)	
N2	0.38262 (12)	0.2660 (3)	0.52490 (7)	0.0219 (4)	
C1	0.37666 (14)	0.2783 (4)	0.69322 (8)	0.0215 (5)	
C2	0.36569 (15)	0.5001 (4)	0.69829 (8)	0.0247 (5)	
H2	0.3618	0.5667	0.7293	0.030*	
C3	0.36066 (16)	0.6208 (4)	0.65619 (9)	0.0250 (5)	
H3	0.3531	0.7742	0.6576	0.030*	
C4	0.36681 (14)	0.5157 (4)	0.61200 (8)	0.0224 (5)	
H4	0.3629	0.5959	0.5827	0.027*	
C5	0.37868 (14)	0.2921 (4)	0.61109 (8)	0.0209 (5)	
C6	0.38647 (15)	0.1703 (4)	0.56563 (8)	0.0221 (5)	
H6	0.3945	0.0171	0.5666	0.027*	
C7	0.38278 (14)	0.1445 (4)	0.48137 (8)	0.0219 (5)	
C8	0.41670 (16)	0.2481 (4)	0.43969 (7)	0.0234 (5)	
H8	0.4428	0.3912	0.4417	0.028*	
C9	0.41266 (17)	0.1437 (4)	0.39534 (8)	0.0272 (5)	
H9	0.4372	0.2151	0.3673	0.033*	
C10	0.37303 (14)	−0.0649 (4)	0.39118 (9)	0.0228 (5)	
C11	0.33938 (16)	−0.1659 (4)	0.43272 (8)	0.0246 (5)	
H11	0.3123	−0.3079	0.4305	0.030*	
C12	0.34413 (15)	−0.0651 (4)	0.47738 (8)	0.0224 (5)	
H12	0.3211	−0.1387	0.5054	0.027*	
C14	0.36452 (17)	−0.1756 (5)	0.34244 (9)	0.0332 (6)	
H14A	0.2966	−0.1623	0.3305	0.050*	
H14B	0.3816	−0.3296	0.3458	0.050*	
H14C	0.4098	−0.1065	0.3195	0.050*	

### Atomic displacement parameters (Å^2^)

**Table:**

	*U*^11^	*U*^22^	*U*^33^	*U*^12^	*U*^13^	*U*^23^
Br1	0.0412 (2)	0.0310 (2)	0.01834 (17)	0.00145 (10)	−0.00218 (9)	0.00303 (9)
N1	0.0239 (10)	0.0212 (11)	0.0203 (10)	0.0009 (7)	−0.0008 (7)	0.0006 (8)
N2	0.0218 (10)	0.0232 (12)	0.0207 (10)	−0.0010 (8)	0.0013 (7)	−0.0021 (8)
C1	0.0207 (11)	0.0236 (13)	0.0202 (11)	−0.0007 (9)	−0.0009 (8)	0.0007 (9)
C2	0.0257 (12)	0.0267 (14)	0.0217 (12)	−0.0008 (9)	0.0000 (9)	−0.0063 (10)
C3	0.0302 (13)	0.0200 (13)	0.0247 (13)	0.0017 (9)	−0.0008 (9)	−0.0018 (10)
C4	0.0251 (11)	0.0218 (14)	0.0203 (12)	0.0004 (9)	0.0011 (8)	0.0012 (10)
C5	0.0180 (11)	0.0243 (13)	0.0203 (11)	0.0000 (9)	0.0000 (7)	0.0003 (10)
C6	0.0234 (11)	0.0196 (13)	0.0234 (12)	0.0022 (9)	−0.0011 (8)	−0.0026 (9)
C7	0.0177 (11)	0.0277 (14)	0.0202 (12)	0.0034 (9)	−0.0005 (8)	−0.0002 (9)
C8	0.0229 (11)	0.0228 (13)	0.0246 (11)	−0.0002 (9)	0.0027 (9)	0.0034 (9)
C9	0.0245 (12)	0.0361 (15)	0.0209 (11)	−0.0004 (10)	0.0042 (9)	0.0034 (10)
C10	0.0182 (11)	0.0300 (14)	0.0201 (12)	0.0019 (9)	−0.0004 (8)	−0.0048 (10)
C11	0.0223 (11)	0.0236 (13)	0.0279 (12)	−0.0002 (9)	−0.0030 (9)	−0.0026 (9)
C12	0.0226 (11)	0.0231 (13)	0.0214 (11)	−0.0010 (9)	−0.0011 (8)	0.0028 (9)
C14	0.0325 (14)	0.0452 (17)	0.0220 (13)	−0.0019 (11)	−0.0001 (9)	−0.0088 (12)

### Geometric parameters (Å, °)

**Table:**

Br1—C1	1.917 (2)	C7—C8	1.394 (3)
N1—C1	1.316 (3)	C7—C12	1.397 (3)
N1—C5	1.351 (3)	C8—C9	1.384 (3)
N2—C6	1.271 (3)	C8—H8	0.9500
N2—C7	1.416 (3)	C9—C10	1.397 (3)
C1—C2	1.380 (3)	C9—H9	0.9500
C2—C3	1.382 (3)	C10—C11	1.382 (3)
C2—H2	0.9500	C10—C14	1.513 (3)
C3—C4	1.384 (3)	C11—C12	1.382 (3)
C3—H3	0.9500	C11—H11	0.9500
C4—C5	1.386 (4)	C12—H12	0.9500
C4—H4	0.9500	C14—H14A	0.9800
C5—C6	1.466 (3)	C14—H14B	0.9800
C6—H6	0.9500	C14—H14C	0.9800
			
C1—N1—C5	116.7 (2)	C12—C7—N2	123.7 (2)
C6—N2—C7	120.5 (2)	C9—C8—C7	120.4 (2)
N1—C1—C2	125.9 (2)	C9—C8—H8	119.8
N1—C1—Br1	115.50 (17)	C7—C8—H8	119.8
C2—C1—Br1	118.63 (17)	C8—C9—C10	121.0 (2)
C1—C2—C3	116.8 (2)	C8—C9—H9	119.5
C1—C2—H2	121.6	C10—C9—H9	119.5
C3—C2—H2	121.6	C11—C10—C9	118.2 (2)
C2—C3—C4	119.2 (2)	C11—C10—C14	120.8 (2)
C2—C3—H3	120.4	C9—C10—C14	121.1 (2)
C4—C3—H3	120.4	C10—C11—C12	121.6 (2)
C3—C4—C5	119.1 (2)	C10—C11—H11	119.2
C3—C4—H4	120.4	C12—C11—H11	119.2
C5—C4—H4	120.4	C11—C12—C7	120.1 (2)
N1—C5—C4	122.2 (2)	C11—C12—H12	119.9
N1—C5—C6	115.7 (2)	C7—C12—H12	119.9
C4—C5—C6	122.1 (2)	C10—C14—H14A	109.5
N2—C6—C5	121.2 (2)	C10—C14—H14B	109.5
N2—C6—H6	119.4	H14A—C14—H14B	109.5
C5—C6—H6	119.4	C10—C14—H14C	109.5
C8—C7—C12	118.7 (2)	H14A—C14—H14C	109.5
C8—C7—N2	117.4 (2)	H14B—C14—H14C	109.5
			
C5—N1—C1—C2	−0.7 (3)	C6—N2—C7—C8	−155.5 (2)
C5—N1—C1—Br1	−179.94 (14)	C6—N2—C7—C12	29.6 (3)
N1—C1—C2—C3	0.7 (3)	C12—C7—C8—C9	−0.5 (3)
Br1—C1—C2—C3	179.93 (15)	N2—C7—C8—C9	−175.66 (19)
C1—C2—C3—C4	−0.1 (3)	C7—C8—C9—C10	1.2 (3)
C2—C3—C4—C5	−0.5 (3)	C8—C9—C10—C11	−1.0 (3)
C1—N1—C5—C4	0.0 (3)	C8—C9—C10—C14	177.4 (2)
C1—N1—C5—C6	−179.88 (17)	C9—C10—C11—C12	0.1 (3)
C3—C4—C5—N1	0.6 (3)	C14—C10—C11—C12	−178.3 (2)
C3—C4—C5—C6	−179.54 (19)	C10—C11—C12—C7	0.6 (3)
C7—N2—C6—C5	−175.22 (17)	C8—C7—C12—C11	−0.4 (3)
N1—C5—C6—N2	179.92 (19)	N2—C7—C12—C11	174.45 (19)
C4—C5—C6—N2	0.0 (3)		
